# Admission risk factors for cerebral vasospasm in ruptured brain arteriovenous malformations: An observational study

**DOI:** 10.1186/cc10345

**Published:** 2011-08-10

**Authors:** Vibol Chhor, Yannick Le Manach, Fréderic Clarençon, Aurélien Nouet, Jean-Louis Daban, Lamine Abdennour, Louis Puybasset, Thomas Lescot

**Affiliations:** 1 Department of Anesthesiology and Critical Care, Groupe Hospitalier Pitié-Salpêtrière, Assistance Publique-Hôpitaux de Paris, Université Pierre et Marie Curie- Paris 6, 47-83 boulevard de l'hôpital, Paris, 75651, France; 2 Centre for Statistics in Medicine - Wolfson College, University of Oxford, Barton Road, Cambridge CB3 9BB, UK; 3 Department of Neuroradiology, Groupe Hospitalier Pitié-Salpêtrière, Assistance Publique-Hôpitaux de Paris, Université Pierre et Marie Curie- Paris 6, 47-83 boulevard de l'hôpital, Paris, 75651, France; 4 Department of Neurosurgery, Groupe Hospitalier Pitié-Salpêtrière, Assistance Publique-Hôpitaux de Paris, Université Pierre et Marie Curie- Paris 6, 47-83 boulevard de l'hôpital, Paris, 75651, France

## Abstract

**Introduction:**

Cerebral vasospasm is a well-documented complication of aneurismal subarachnoid hemorrhage but has not been extensively studied in brain arteriovenous malformations (BAVMs). Here, our purpose was to identify risk factors for cerebral vasospasm after BAVM rupture in patients requiring intensive care unit (ICU) admission.

**Methods:**

Patients admitted to our ICU from January 2003 to May 2010 for BAVM rupture were included in this observational study. Clinical, laboratory and radiological features from admission to ICU discharge were recorded. The primary endpoint was cerebral vasospasm by transcranial Doppler (TCD-VS) or cerebral infarction (CI) associated with vasospasm. Secondary endpoints included the Glasgow Outcome Scale (GOS) at ICU discharge.

**Results:**

Of 2,734 patients admitted to our ICU during the study period, 72 (2.6%) with ruptured BAVM were included. TCD-VS occurred in 12 (17%) and CI in 6 (8%) patients. All patients with CI had a previous diagnosis of TCD-VS. A Glasgow Coma Scale score <8 was a risk factor for both TCD-VS (relative risk (RR), 4.7; 95% confidence interval (95% CI), 1.6 to 26) and CI (RR, 7.8; 95% CI, 0.1 to 63). Independent risk factors for TCD-VS by multivariate analysis were lower Glasgow Coma Scale score (odds ratio (OR) per unit decrease, 1.38; 95% CI, 1.13 to 1.80), female gender (OR, 4.86; 95% CI, 1.09 to 25.85), and younger age (OR per decade decrease, 1.39; 95% CI, 1.05 to 1.82). The risk of a poor outcome (GOS <4) at ICU discharge was non-significantly increased in the patients with TCD-VS (RR, 4.9; 95% CI, 0.7 to 35; *P *= 0.09). All six patients with CI had poor outcomes.

**Conclusions:**

This is the first cohort study describing the incidence and risk factors for cerebral vasospasm after BAVM rupture. Larger studies are needed to investigate the significance of TCD-vasospasm and CI in these patients.

## Introduction

Cerebral vasospasm has been extensively studied following aneurismal subarachnoid hemorrhage (SAH) and has also been reported after traumatic brain injury [1] or neurosurgery [2]. After aneurismal SAH, several risk factors present at admission have been identified, such as younger age, cigarette smoking, poor clinical grade, arterial hypertension, intracerebral hemorrhage, and thick cisternal clot [3-5].

Although rupture of a brain arteriovenous malformation (BAVM) is a cause of SAH, few data are available on the incidence of cerebral vasospasm after BAVM rupture. In a series of 100 patients admitted between 1957 and 1977, Parkinson *et al*. [6] found a single case of symptomatic vasospasm. In recent years, however, transcranial Doppler (TCD) and CT/MRI cerebral angiography have contributed to improve the detection of vasospasm. Severe vasospasm associated with delayed cerebral infarction (CI) was reported recently in young adults [7-11] and children [10,12,13] with BAVM rupture. Medical treatments may be effective in minimizing the adverse consequences of vasospasm and improving outcomes after aneurismal SAH [14]. These treatments may also be effective in ruptured BAVM. Early vasospasm detection in patients with ruptured BAVM would allow evaluations of therapeutic interventions such as calcium-channel blockers and triple-H therapy. The identification of risk factors for vasospasm would be expected to assist in early vasospasm detection.

Here, our aim was to identify risk factors for cerebral vasospasm present at admission to the intensive care unit (ICU) for intracerebral bleeding following BAVM rupture.

## Materials and methods

This observational study was conducted in compliance with STROBE (Strengthening the Reporting of Observational Studies in Epidemiology) guidelines [15], with the slight adjustments detailed below.

### Patients

Consecutive patients admitted to our 25-bed neurosurgical ICU with ruptured BAVM from January 2003 to May 2010 were eligible. BAVM rupture was defined as SAH, intraventricular hemorrhage (IVH), or intracerebral hematoma visualized on the admission computed tomography (CT) scan with concomitant BAVM visualization by digital subtraction angiography (DSA) or CT-angiography. Exclusion criteria were admission more than four days after BAVM rupture suggesting suboptimal initial care, death within four days after BAVM rupture (minimal time to vasospasm), BAVM rupture after elective treatment, and age younger than 15 years. For this single-center retrospective observational study using anonymized information, informed consent was waived by our local ethics review board (Comité de Protection des Personnes - Ile de France VI Pitié-Salpêtrière) and according to the French law (Act n°78-17 of 6 January 1978 on data processing, data files, and individual liberties).

### Clinical management

The timing and type of treatment (embolization, surgical resection, or both) were decided by consensus between the neurosurgeon and interventional neuroradiologist based on the clinical presentation and on the location, size, and angioarchitecture of the BAVM. All patients were admitted to the ICU. None received prophylactic nimodipine or statin therapy. A central venous line and an arterial catheter were inserted when required. Intravenous isotonic saline was given routinely to maintain normovolemia. After the BAVM was secured, systolic arterial blood pressure was maintained above 130 to 140 mmHg, if needed by continuously infusing norepinephrine. Intracranial pressure (ICP) elevation was treated by cerebrospinal fluid drainage, mechanical ventilation, reinforced sedation, and, rarely, moderate hypothermia. CT was performed regularly during the ICU stay, routinely on the day of transfer from the ICU to the ward, and in the event of clinical deterioration, to look for secondary complications such as hydrocephalus, re-bleeding, or ischemia. Patients diagnosed with TCD vasospasm (TCD-VS) were treated with continuous intravenous nimodipine (2 mg/h) and, if the BAVM was secured, continuous norepinephrine infusion for arterial blood pressure elevation. DSA was performed in transportable patients. Selective intraarterial chemical vasodilation (nimodipine) and transarterial balloon dilation were considered to be second-line treatments in patients with secured BAVMs.

### Study variables

At admission, we recorded factors describing the population and factors potentially associated with outcomes, including age, gender, smoking history, arterial hypertension, diabetes, and Glasgow Coma Scale (GCS) score.

The consequences of BAVM rupture identified on the admission cerebral CT scan were recorded as intraventricular hemorrhage, intracerebral hematoma, and/or SAH. SAH was classified as diffuse (diffuse deposition or thin layer of blood <1 mm), focal (localized clot >1 mm), or absent. A neuroradiologist (FC) examined the DSA images to determine the BAVM angioarchitectural features including location; size; venous drainage pattern; and presence of a Willis, intranidal, or feeding-vessel aneurism. The Spetzler-Martin grade [16] based on nidus size, venous drainage pattern, and neurological eloquence of adjacent brain (from 1 to 5, with a higher grade indicating a higher risk of surgical complication) was also recorded. BAVM treatments such as surgical BAVM resection, embolization (microcatheter arterial occlusion using occlusive materials), or both were recorded. Patients with incomplete BAVM treatment were identified. Early BAVM treatment was defined as treatment started within the first seven days after hospital admission. Intracranial hypertension was defined as ICP greater than 20 mmHg for more than 10 minutes.

### Endpoints

The primary endpoint was vasospasm in the ICU, with vasospasm defined as either TCD-VS or CI. Transcranial color-coded Doppler sonography (Envisor, Philips Medical Systems, Bothell, WA, USA) was performed daily by a neurointensivist in unconscious patients and in awake patients with symptoms (deteriorating consciousness, focal deficit, headache, fever, confusion) as part of the routine screening protocol used in our ICU. TCD-VS was defined as blood flow velocity >120 cm/s in any cerebral vessel [4,17,18]. Velocities were measured at a distance from the BAVM visualized by color-coded sonography, and the Aaslid index was determined to exclude a hyperemia-induced velocity increase [19]. Cerebral infarction was defined as CT or MRI evidence of cerebral infarction associated with vasospasm with no other identifiable cause [20].

The Glasgow Outcome Scale (GOS) at ICU discharge was among the secondary endpoints. We considered two categories: poor outcome (death (GOS = 1), vegetative state (GOS = 2) or severe disability (GOS = 3)), and good outcome (moderate disability (GOS = 4) or good recovery (GOS = 5)). Length of stay was the time from admission to discharge in survivors. Finally, to take early deaths into account, we recorded ICU-free days as the number of days spent outside the ICU within the first 40 days; patients who died at any time were classified as having no ICU-free days.

### Statistical analyses

Data are expressed as mean with standard deviation for normal quantitative variables, median with the interquartile range (IQR) for non-normal quantitative variables, and numbers (percentages) for qualitative variables. Normality was assessed using the D'Agostino-Pearson omnibus test. The unpaired Student's *t *test was used to compare means, the Mann-Whitney U test to compare medians, and Fisher's exact method to compare proportions.

Stepwise logistic regression was performed to identify risk factors for TCD-VS. We used a semi-parsimonious approach, including only the available unbiased variables (Table 1). Discrimination of the final models was assessed using the c-statistic and calibration using the Hosmer-Lemeshow statistic. Internal validation was performed using 10-fold cross-validation [21] and was described based on the difference (optimism) between the c-statistic in the overall population and cross-validation samples and on the optimism-corrected c-statistic. The number of patients with CI was too small for a separate multivariate analysis of risk factors for this event. *P-*values were two-tailed and *P-*values less than 0.05 were considered significant. Statistical analysis was performed using R software and specific packages [22].

**Table 1:** Associations linking transcranial Doppler vasospasm and cerebral infarction to admission characteristics and outcomes

	All Patients N = 72	TCD-VS N = 12			CI N = 6		
		**N (%)**	**RR (95% CI)**	***P***	**N (%)**	**RR (95% CI)**	***P***
**Demographics and medical history**							
Age (years)	40 +/- 9	33 +/-10	-	0.05	38 +/- 13	-	0.98
Female sex	29 (40%)	7 (58%)	2.1 (0.7 to 5.9)	0.20	4 (67%)	2.9 (0.6 to 15.1)	0.22
Smokers	18 (25%)	1 (8%)	0.3 (0.03 to 2.0)	0.27	1 (17%)	0.6 (0.07 to 4.8)	0.99
Hypertension	8 (11%)	0 (0%)	-	0.34	0 (0%)	-	0.99
Diabetes	2 (3%)	0 (0%)	-	0.99	0 (0%)	-	0.99
**Admission features**							
GCS score <8	28 (39%)	9 (75%)	4.7 (1.6 to 26)	0.001	5 (83%)	7.8 (0.1 to 63)	0.03
Seizure	9 13%)	2 (17%)	1.4 (0.4 to 5.4)	0.63	2 (33%)	3.5 (0.7 to 16.4)	0.16
Troponin elevation	18 (25%)	4 (33%)	1.5 (0.5 to 4.4)	0.48	1 (17%)	0.6 (0.07 to 4.8)	0.99
EKG abnormality	2 (3%)	1 (8%)	3.2 (0.7 to 14)	0.31	0 (0%)	-	0.99
ICH	64 (89%)	10 (83%)	0.6 (0.2 to 2.4)	0.61	4 (67%)	0.2 (0.05 to 1.2)	0.14
IVH	54 (75%)	10 (83%)	1.7 (0.4 to 6.9)	0.72	6 (100%)	-	0.33
SAH	32 (44%)	7 (58%)	1.7 (0.6 to 5.0)	0.34	3 (50%)	1.2 (0.3 to 5.8)	0.99
*Diffuse*	14 (19%)	4 (33%)			1 (17%)		
*Focal*	18 (25%)	3 (25%)	-	0.40	2 (33%)	-	0.85
*Absent*	40 (56%)	5 (42%)			3 (50%)		
**BAVM characteristics and treatment**							
BAVM location							
*Frontal*	25 (35%)	5 (43%)			4 (67%)		
*Temporal*	10 (14%)	0 (0%)			0 (0%)		
*Parietal*	15 (21%)	4 (33%)	-	0.31	1 (17%)	-	0.77
*Occipital*	8 (11%)	0 (0%)			0 (0%)		
*Posterior fossa *	14 (19%)	3 (25%			1 (17%)		
Size of BAVM*							
<3 cm	43 (63%)	7 (58%)			5 (83%)		
3 to 6 cm	19 (28%)	3 (25%)	-	0.52	0 (0%)	-	0.29
>6 cm	6 (8%)	2 (17%)			1 (17%)		
Adjacent to eloquent brain *	50 (69%)	7 (58%)	0.5 (0.2 to 1.4)	0.27	3 (50%)	0.4 (0.07; 1.6)	0.32
Deep venous drainage*	31 (43%)	7 (58%)	1.7 (0.5 to 4.7)	0.36	4 (67%)	2.4 (0.5 to 12)	0.40
Co-existing aneurysm*							
None	50 (73%)	11 (92%)			5 (83%)		
Intranidal	8 (12%)	1 (8%)	-	0.99	1 (17%)	-	0.99
Feeding vessel	8 (12%)	0 (0%)			0 (0%)		
Willis	2 (3%)	0 (0%)			0 (0%)		
Surgical resection	35 (49%)	6 (50%)	1.0 (0.3 to 2.8)	0.99	2 (33%)	0.5 (0.1 to 2.5)	0.67
Embolization	15 (20%)	2 (17%)	0.7 (0.2 to 2.9)	0.99	1 (17%)	0.7 (0.1 to 5.6)	0.99
Mixed	6 (8%)	0(0%)	to	0.58	0 (0%)	-	0.99
Complete treatment	34 (47%)	5 (42%)	1.6 (0.5 to 4.4)	0.53	3 (50%)	1.2 (0.2 to 4.2)	0.99
Early treatment	43 (60%)	8 (67%)	1.4 (0.4 to 4.1)	0.75	3 (50%)	0.7 (0.1 to 3.1)	0.67
**ICU outcomes**							
Norepinephrine	46 (64%)	11 (92%)	6.2 (0.8 to 45)	0.04	6 (100%)	-	0.08
Intracranial hypertension	41 (57%)	10 (83%)	3.7 (0.9 to 16)	0.06	6 (100%)	3.8 (0.5 to 31)	0.22
Hypothermia	15 (21%)	4 (33%)	1.9 (0.7 to 5.5)	0.26	3 (50%)	3.8 ( 0.8 to 17)	0.10
CSF drainage	42 (59%)	10 (83%)	3.5 (0.8 to 15)	0.10	6 (100%)	-	0.04
ICU LOS	28 (12 to 38)	34 (17 to 41)	-	0.09	27 (14 to 36)	-	0.74
ICU-free days	6 (0 to 28)	1 (0 to 3)	-	0.01	0 (0 to 2)	-	0.06
Unfavorable outcome (GOS 1-3)	50 (69%)	11 (92%)	4.9 (0.7 to 35)	0.09	6 (100%)	-	0.17

ICU mortality	15 (21%)	5 (42%)	2.7 (1.0 to 7.3)	0.11	3 (50%)	3.8 (0.8 to 17)	0.10

BAVM, brain arteriovenous malformation; CI, cerebral infarction, CSF, cerebrospinal fluid; GCS, Glasgow Coma Scale; ICH, intracerebral hemorrhage; ICU, intensive care unit; IVH, intraventricular hemorrhage; LOS, length of stay; RR (95%CI), relative risk with the 95% confidence interval; SAH, subarachnoid hemorrhage; TCD-VS, transcranial Doppler vasospasm. * n = 68 patients (data missing in four patients without vasospasm)

Values are median (IQR) for quantitative variables and N (%) for qualitative variables.

## Results

### Cohort description

**Figure 1:**
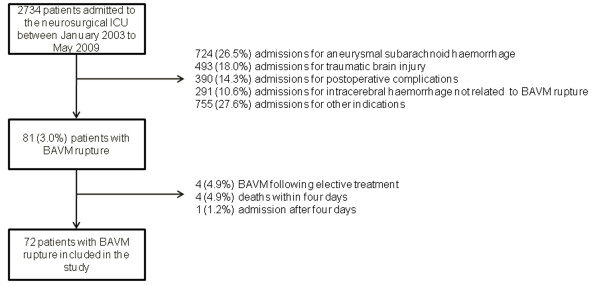
**Flow chart of the study patients**. BAVM, brain arteriovenous malformation; ICU, intensive care unit.

Figure 1 shows the patient flowchart. During the seven-year study period, 2,734 patients were admitted to our neurosurgical ICU including 81 (3.0%) with BAVM rupture. Of these 81 patients, 9 were excluded, for the following reasons: BAVM rupture during elective treatment (n = 4), death before Day 4 (n = 4), or admission more than four days after BAVM rupture (n = 1). This left 72 patients for the study.

**Figure 2:**
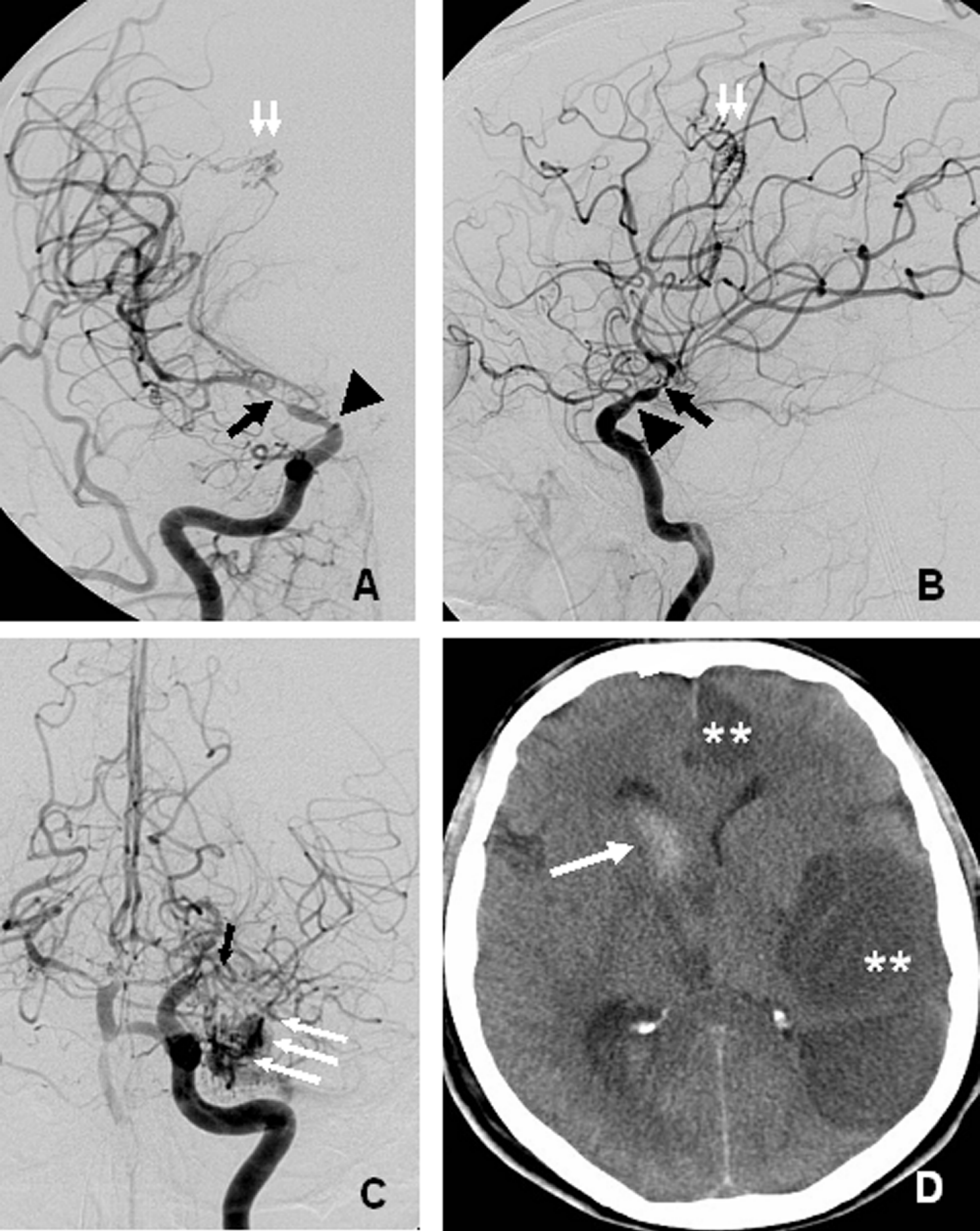
**Cerebral digital subtraction angiography in a patient with vasospasm after rupture of a BAVM**. Cerebral digital subtraction angiography performed 11 days after intraventricular hemorrhage in a 33-year-old patient with two brain arteriovenous malformations (BAVMs): a ruptured BAVM in the right frontal lobe and an intact BAVM in the left temporal lobe. Right internal carotid artery (ICA) injection, anteroposterior (AP) (**A**) and lateral (**B**) views: severe vasospasm of the M1 segment of the right middle cerebral artery (black arrow) and terminal right internal carotid artery (black arrowhead). Small frontal BAVM (double white arrow). Left ICA injection, AP view (**C**): severe vasospasm of the M1 segment (black arrow). The other BAVM is visible in the temporal lobe (triple white arrow). Cerebral CT scan (**D**): vasospasm-associated cerebral infarction in both the left middle and the left anterior cerebral arteries (**). Note the remnant of the intraventricular hemorrhage (white arrow).

Of the 72 study patients, 12 (17%) had TCD-VS and 6 (8%) had CI. DSA was performed in 4 of the 12 TCD-VS patients and showed diffuse vasospasm in all of them. Figure 2 shows an example of BAVM and angiographic cerebral vasospasm associated with CI.

**Figure 3:**
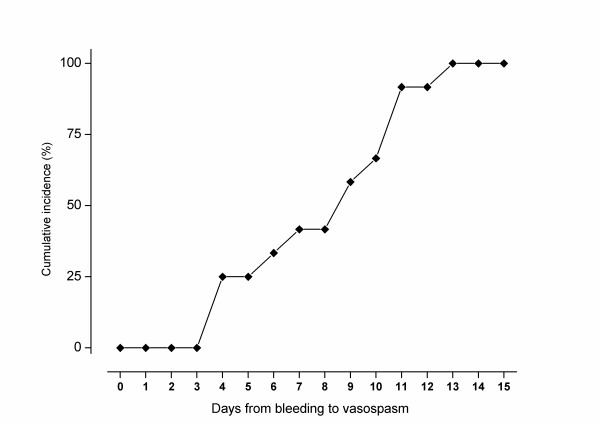
**Cumulative incidence of transcranial Doppler vasospasm in patients with ruptured brain arteriovenous malformations**.

Median time from BAVM rupture to TCD-VS diagnosis was nine days (IQR, 4 to 11). Figure 3 reports the cumulative incidence of TCD-VS according to time from BAVM rupture to TCD-VS diagnosis. All patients with CI had a previous diagnosis of TCD-VS.

### Admission risk factors for vasospasm

Table 1 lists the admission features and ICU outcomes of patients diagnosed with TCD-VS and CI. The risk of developing TCD-VS was greater in young patients (*P *= 0.05) and in patients with GCS scores <8 (*P *< 0.01). Neither TCD-VS nor CI was significantly associated with the amount of subarachnoid blood, intraventricular hemorrhage, or intracerebral hematoma. SAH was diagnosed in 32 (44%) of the 72 study patients, including 7 (58%) of the 12 patients with TCD-VS and 3 (50%) of the 6 patients with CI. IVH was present in 54% of the patients. More specifically, isolated IVH was diagnosed in 7 of the 72 patients, including 6 of the 60 patients without vasospasm and 1 patient with TCD-VS and CI. Neither TCD-VS nor CI was associated with BAVM location, angioarchitectural features or treatment modalities. The risk of developing CI was greater in patients with GCS scores <8 (*P *= 0.03). Details on the clinical and radiological features and treatment of each patient with cerebral vasospasm are given in Table 2.

**Table 2:** Cases of cerebral vasospasm after rupture of a BAVM, adapted from Yanaka *et al*. [3] and Gerard *et al*. [7]

	Clinical and radiological features					BAVM angioarchitectural features						BAVM treatment				Vasospasm features and management						Outcome
#	Sex	GCS	SAH	IVH	ICH	Location	Venous drainage	Size (mm)	Eloquence	Spetzler Martin Grade	Co-existing aneurism	BAVM treatment	Exclusion	Time to treatment	Time to onset (days)	TCD Mean flow velocity (cm/s)	TCD-VS artery	CI location	Medical treatment	Endovascular treatment	GOS	Causes of death
1	M	4	A	Y	N	R. frontal	Superf.	<30	N	1	N	None	-	-	11	200	B MCA	L ACA L MCA	Nimodipine	IA Nimodipine	1	Cerebral ischemia
2	F	3	F	Y	Y	R. temporal	Superf.	30 to 60	N	2	N	Surgery	Total	<24 h	9	160	B MCA		None	None	2	-
3	M	3	F	Y	Y	R. frontal	Deep	>60	Y	5	N	None	-	-	10	150	B MCA	R MCA R ACA L MCA	None	None	1	Intracranial hypertension
4	F	3	D	Y	Y	Cerebellum	Superf.	<30	Y	2	N	Surgery	Total	<24 h	6	169	B MCA R ACA L VA, BA		None	None	1	Intracranial hypertension
5	M	10	A	N	Y	Cerebellum	Superf.	<30	Y	2	N	Surgery	Total	<24 h	4	150	B MCA B ACA		Nimodipine	None	2	-
6	M	4	D	Y	Y	R. parietal	Deep	30 to 60	N	3	N	Surgery	Total	<24 h	7	222	L MCA		Nimodipine	IA Nimodipine IA Milrinone	1	Initial bleed
7	F	7	A	Y	Y	L. parietal	Deep	<30	N	2	Intranidal	None	-	-	13	165	B MCA	R MCA	Nimodipine	None	3	-
8	F	7	F	Y	Y	Posterior cranial fossa	Superf.	<30	N	1	N	Surgery	Total	<24 h	11	162	B MCA	B MCA B PCA	None	IA Nimodipine IA Milrinone	1	Cerebral ischemia
9	F	13	D	Y	N	L. frontal	Deep	<30	Y	3	N	Embolization	Total	5 d	9	160	R MCA	R MCA RACA	Nimodipine	IA Nimodipine IA Milrinone	2	-
10	F	5	A	Y	Y	R. frontal	Deep	<30	Y	3	N	Surgery	Total	<24 h	4	194	B MCA R ACA	L MCA	Nimodipine	None	3	-
11	F	3	D	Y	Y	R. frontal	Deep	>60	Y	5	N	Embolization	Incom-plete	<24 h	11	185	B MCA B ACA		Nimodipine	None	5	-
12	M	8	A	N	Y	R. parietal	Deep	30 to 60	Y	4	N	None	-	-	4	217	R MCA		Nimodipine	None	3	-

A, absent; ACA, anterior cerebral artery; B, bilateral; BA, basilar artery; BAVM, brain arteriovenous malformation; CI, cerebral infarction; D, diffuse; F, focal; GCS, Glasgow Coma Scale; GOS, Glasgow Outcome Scale; IA, intraarterial; ICA, internal carotid artery; ICH, intracerebral hemorrhage; IVH, intraventricular hemorrhage; L, left; MCA, middle cerebral artery; N, no; PCA, posterior cerebral artery; R, right; SAH, subarachnoid hemorrhage; Superf, superficial; TCD, transcranial Doppler; TCD-VS, vasospasm detected using transcranial Doppler; VA, vertebral artery; Y, yes.

By multivariate analysis, three factors were associated with TCD-VS, namely, a worse GCS score, female gender, and younger age (Table 3). The final model had good discrimination (c-statistic = 0.82) and calibration (Hosmer-Lemeshow statistic *P-*value = 0.16). The internal validation procedure showed good robustness of the final model (optimism = 0.04).

**Table 3:** Factors present at admission and associated with transcranial Doppler vasospasm by multivariate analysis

	Odds ratio	95% confidence interval	*P-*value
Glasgow Coma Scale *(per unit decrease)*	1.38	(1.13 to 1.80)	0.005
Female gender	4.86	(1.09 to 25.85)	0.04
Age *(per 10-year decrease) *	1.39	(1.05 to 1.82)	0.01

C-statistic = 0.82; 10-fold cross-validation optimism = 0.03; corrected c-statistic = 0.79; Hosmer-Lemeshow statistic = 1.39; *P *= 0.16 (df = 9).

### Consequences of cerebral vasospasm

Patients with TCD-VS had significantly fewer ICU-free days. Of the 72 study patients, 50 had poor outcomes (GOS 1, 2 or 3) at ICU discharge. The poor outcome was directly ascribable to the initial bleed in 42 patients, to CI in 6 patients, and to re-bleeding in 2 patients. The risk of a poor outcome was non-significantly increased in the patients who developed TCD-VS (relative risk, RR, 4.9; 95% confidence interval 95% CI, 0.7 to 35; *P *= 0.09). All six patients with CI had poor outcomes. In the patients without vasospasm, ICU mortality was 17% and causes of death were as follows: initial bleed (n = 5), refractory intracranial hypertension (n = 3), and re-bleeding (n = 2). Of the patients with TCD-VS, 42% died and death was considered directly related to CI in two patients, intracranial hypertension in two patients, and the initial bleed in one patient (Table 2). Using logistic regression including age, GSC, TCD and CI, the only independent factor for poor outcome was GCS (odds ratio per unit increase, 0.82; 95% CI, 0.70 to 0.94; *P *= 0.01) whereas TCD-VS was not (OR, 2.80; 95%CI, 0.41 to 51; *P *= 0.36).

## Discussion

To the best of our knowledge, this is the first cohort study describing the incidence and risk factors for cerebral vasospasm after BAVM rupture. Although rare (3.0% of admissions to our ICU), BAVM rupture was complicated by vasospasm within a few days in 17% of patients (TCD-VS) or 8% of patients (CI) depending on the definition used. In our study, the prevalence of cerebral vasospasm was lower than in studies of the main other causes of cerebral vasospasm. TCD-VS was diagnosed in 31% to 70% of patients after aneurismal SAH [4,23] and in 20% to 50% patients with traumatic brain injury [1,24-26]. We found that time to TCD-VS was 4 to 11 days in 92% of patients (median, 9 days). In the case-reports of BAVM rupture published over the last two decades, median time from bleeding to vasospasm was 13 days [7-13].

Similarly, in a study of 50 patients, angiographic cerebral vessel narrowing was noted 3 to 12 days after BAVM rupture [27]. Furthermore, the time to vasospasm in our study of BAVM rupture was comparable to that reported after SAH [28] and traumatic brain injury [1].

We identified three early risk factors for vasospasm: age, gender, and GCS score. All three factors can be easily assessed at admission, which may help to stratify patients presenting with BAVM rupture. Interestingly, although no previous studies are available for comparison, there are eight published case-reports of vasospasm after BAVM rupture, all in young patients (mean age was 26 ± 12 years), six of whom are females [7-13]. This is in accordance with our finding that female gender and younger age were associated with vasospasm. The third independent risk factor for vasospasm in our study was a lower GCS score. Similarly, in previous studies, lower levels of consciousness predicted vasospasm after SAH [29] and traumatic brain injury [1]. In the present study, intraventricular hemorrhage was present in 83% and 100% of the patients with TCD-VS and CI, respectively, in accordance with the occurrence of IVH in all eight previously reported cases of vasospasm following BAVM rupture. The amount of blood in the subarachnoid spaces as assessed by the Fisher score has been recognized as a strong predictor of cerebral vasospasm after SAH [5]. However, the Fisher score did not significantly predict cerebral vasospasm in our study, and vasospasm has been reported after BAVM rupture without SAH [7,8,10,13]. Although the exact pathophysiology of cerebral vasospasm remains unknown, our data and previously published cases suggest that IVH, but not SAH, may be associated with the development of vasospasm after BAVM rupture. Experimental evidence suggests that oxyhemoglobin release secondary to blood clot elimination may initiate the cascade involving vasoactive substances such as endothelium-derived nitrite oxide and endothelin-1, which leads to cerebral vasospasm [30,31]. Further research is needed to clarify the pathophysiology of vasospasm after BAVM rupture and to explain the different impacts of subarachnoid and intraventricular clots on the genesis of vasospasm. Due to the small number of patients included in the present study, we were not able to identify additional predictors, although we found a trend toward an association between angio-architectural BAVM features and cerebral vasospasm. The high proportion of patients who received norepinephrine in the TCD-VS and CI groups compared to the group without vasospasm is ascribable to our policy of inducing arterial blood pressure elevation in patients diagnosed with vasospasm, as part of "triple-H" therapy.

Although evidence is lacking that treatments such as triple-H therapy, transluminal balloon angioplasty, or selective intraarterial vasodilator infusion are effective in SAH patients, these strategies are commonly used in this group of patients. Based on the current data it would be worthwhile to investigate the efficacy of these treatments in patients with ruptured BAVMs. No clear recommendations about arterial blood pressure management after BAVM treatment are available. Hyperemia has been documented after BAVM treatment. Although the underlying mechanism seems unrelated to systemic hemodynamic changes [32], induced moderate hypertension may cause cerebral and systemic complications. Nevertheless, preventive strategies, such as nimodipine, might deserve evaluation in patients with BAVM rupture who are at high risk for vasospasm.

CI has been identified as the only outcome predictor in patients with SAH [4]. In the present study, TCD-VS and CI were associated with a non-significant increase in the risk of poor outcomes. Although the 21% death rate found in our study is close to the 18% 30-day rate reported by Brown *et al*. [33], it was lower than the 29% rate found by the same group in patients with previously untreated BAVM [34]. Nevertheless, the Colombia group found a lower mortality rate [35] and another study found no mortality at all [36]. Furthermore, in a defined-population study, the case-fatality rate in patients younger than 60 years was about 10% [37]. The comparatively high mortality rate in our patients may be ascribable to differences in severity at admission. In our series, all the patients required ICU admission and 40% were comatose. Since no high-level evidence exists concerning the management of unruptured BAVM, heterogeneity in the treatment methods may contribute to explain mortality rate differences across studies. The ongoing Randomized Trial of Unruptured Brain Arteriovenous Malformations (ARUBA) [38] comparing treatment versus conservative management of unruptured BAVM can be expected to provide answers on this last point.

Unfortunately, the number of patients included was too small to determine whether TCD-VS and CI were independently associated with a poor outcome. Several definitions of cerebral vasospasm are commonly used, including TCD velocity elevation above 120 cm/s, symptomatic vasospasm, angiographic vasospasm, and CI diagnosed by CT or MRI. TCD is a well-validated tool for detecting vasospasm [39,40] with acceptable positive and negative predictive values for angiographic vasospasm but low sensitivity for predicting the neurological outcome [41]. Moreover, intra- and inter-observer variability is of concern and should be taken into account when interpreting velocity changes over time. Nevertheless, intra-observer bias may be minimized by having the same neurointensivist perform all TCD investigations in a given patient [42], as was the case in the present study. Furthermore, mean flow velocities were well above 120 cm/s, and using a higher cut-off point of 150 cm/s would not have changed our results. In our study, TCD was performed in unconscious patients and in awake patients with symptoms. Since not all patients with vasospasm have symptoms, this approach may have underestimated the true incidence of TCD-VS. Angiographic vasospasm was not considered in our study. The absence of recommendations about vasospasm management after BAVM rupture and the poor clinical condition of some patients precluding transport to the radiology department explain that DSA was not performed routinely. This weakness of our study is mitigated by the good reported correlation between TCD and DSA [39] for vasospasm assessment. Furthermore, DSA may require general anesthesia and is associated with a small risk of procedure-related stroke. Finally, no treatment recommendations are available for BAVM rupture with vasospasm. Conceivably, the local cerebral blood flow modifications induced by intra-arterial treatments may lead to re-bleeding, especially when the BAVM has not been secured.

There are several limitations to our study. First, we used a retrospective design in a small number of patients from a single center. However, given the prevalence of BAVM of only about 0.01% in the general population [43], a retrospective design was appealing to ensure study completion within a reasonable timeframe. Second, our data from a single center may not apply to all other centers. Third, vasospasm following BAVM rupture is rare and, consequently, our sample size was small, limiting the statistical power of our study, which may have led us to miss a number of risk factors. Moreover, the number of patients included in the present study was too small to investigate properly whether TCD-VS and CI were independent predictors of a poor outcome. This crucial point will have to be determined in a larger study. In addition, the number of patients with CI was also too small for a separate multivariate analysis of risk factors for this event.

## Conclusions

After BAVM rupture, TCD-VS occurred in 17% of patients and CI in 8%. Admission risk factors for TCD-VS were low GCS, younger age, and female gender. A non-significant trend toward poorer outcome exists in patients with TDC-VS and CI. Prospective, multicenter studies are needed to further assess the incidence and significance of vasospasm and CI after BAVM rupture and to identify additional predictors.

## Key messages

• Transcranial Doppler cerebral vasospasm is a common complication following brain arteriovenous rupture.

• Cerebral vasospasm in brain arteriovenous rupture is associated with low GCS, young age, and female gender.

## Abbreviations

ARUBA: Randomized Trial of Unruptured Brain Arteriovenous Malformations; BAVM: brain arteriovenous malformation; CI: cerebral infarction; CT: Computed Tomography; GCS: Glasgow Coma Scale; GOS: Glasgow Outcome Scale; DSA: Digital Subtraction Angiography; ICP: intracranial pressure; ICU: intensive care unit; IVH: intraventricular hemorrhage; IQR: interquartile range; MRI: Magnetic Resonance Imaging; OR: odds ratio; RR: relative risk; SAH: subarachnoid hemorrhage; STROBE: Strengthening the Reporting of Observational Studies in Epidemiology; TCD-VS: Transcranial Doppler vasospasm.

## Competing interests

The authors declare that they have no competing interests.

## Authors' contributions

VC acquired the data and drafted the manuscript. YLM performed the statistical analysis and helped to draft the manuscript. FC, AN and LA participated in the study design and helped to draft the manuscript. JLD helped to acquire the data and to draft the manuscript. LP conceived and designed the study and helped to draft the manuscript. TL conceived and designed the study, acquired the data, and wrote the manuscript. All authors read and approved the final manuscript.

## References

[B1] Oertel M Boscardin WJ Obrist WD Glenn TC McArthur DL Gravori T Lee JH Martin NA Posttraumatic vasospasm: the epidemiology, severity, and time course of an underestimated phenomenon: a prospective study performed in 299 patients J Neurosurg 2005 103 812 824 10.3171/jns.2005.103.5.0812 16304984

[B2] Aoki N Origitano TC al-Mefty O Vasospasm after resection of skull base tumors Acta Neurochir (Wien) 1995 132 53 58 10.1007/BF01404848 7754859

[B3] Claassen J Bernardini GL Kreiter K Bates J Du YE Copeland D Connolly ES Mayer SA Effect of cisternal and ventricular blood on risk of delayed cerebral ischemia after subarachnoid hemorrhage: the Fisher scale revisited Stroke 2001 32 2012 2020 10.1161/hs0901.095677 11546890

[B4] Frontera JA Fernandez A Schmidt JM Claassen J Wartenberg KE Badjatia N Connolly ES Mayer SA Defining vasospasm after subarachnoid hemorrhage: what is the most clinically relevant definition? Stroke 2009 40 1963 1968 10.1161/STROKEAHA.108.544700 19359629

[B5] Fisher CM Kistler JP Davis JM Relation of cerebral vasospasm to subarachnoid hemorrhage visualized by computerized tomographic scanning Neurosurgery 1980 6 1 9 10.1227/00006123-198001000-00001 7354892

[B6] Parkinson D Bachers G Arteriovenous malformations. Summary of 100 consecutive supratentorial cases J Neurosurg 1980 53 285 299 10.3171/jns.1980.53.3.0285 7420143

[B7] Gerard E Frontera JA Wright CB Vasospasm and cerebral infarction following isolated intraventricular hemorrhage Neurocrit Care 2007 7 257 259 10.1007/s12028-007-0057-1 17522787

[B8] Kobayashi M Takayama H Mihara B Kawase T Severe vasospasm caused by repeated intraventricular haemorrhage from small arteriovenous malformation Acta Neurochir (Wien) 2002 144 405 406 10.1007/s007010200059 12021892

[B9] Kothbauer K Schroth G Seiler RW Do DD Severe symptomatic vasospasm after rupture of an arteriovenous malformation AJNR Am J Neuroradiol 1995 16 1073 1075 7639129 PMC8337792

[B10] Maeda K Kurita H Nakamura T Usui M Tsutsumi K Morimoto T Kirino T Occurrence of severe vasospasm following intraventricular hemorrhage from an arteriovenous malformation. Report of two cases J Neurosurg 1997 87 436 439 10.3171/jns.1997.87.3.0436 9285611

[B11] Yokobori S Watanabe A Nakae R Onda H Fuse A Kushimoto S Yokota H Cerebral vasospasms after intraventricular hemorrhage from an arteriovenous malformation: case report Neurol Med Chir (Tokyo) 2010 50 320 323 10.2176/nmc.50.320 20448426

[B12] Pendharkar AV Guzman R Dodd R Cornfield D Edwards MS Successful treatment of severe cerebral vasospasm following hemorrhage of an arteriovenous malformation. Case report J Neurosurg Pediatr 2009 4 266 269 10.3171/2009.4.PEDS09126 19772412

[B13] Yanaka K Hyodo A Tsuchida Y Yoshii Y Nose T Symptomatic cerebral vasospasm after intraventricular hemorrhage from ruptured arteriovenous malformation Surg Neurol 1992 38 63 67 10.1016/0090-3019(92)90214-8 1615376

[B14] Zwienenberg-Lee M Hartman J Rudisill N Muizelaar JP Endovascular management of cerebral vasospasm Neurosurgery 2006 59 S139 147 discussion S3-13 17053596 10.1227/01.NEU.0000239252.07760.59

[B15] von Elm E Altman DG Egger M Pocock SJ Gotzsche PC Vandenbroucke JP Strengthening the Reporting of Observational Studies in Epidemiology (STROBE) statement: guidelines for reporting observational studies BMJ 2007 335 806 808 10.1136/bmj.39335.541782.AD 17947786 PMC2034723

[B16] Spetzler RF Martin NA A proposed grading system for arteriovenous malformations J Neurosurg 1986 65 476 483 10.3171/jns.1986.65.4.0476 3760956

[B17] Lysakowski C Walder B Costanza MC Tramer MR Transcranial Doppler versus angiography in patients with vasospasm due to a ruptured cerebral aneurysm: A systematic review Stroke 2001 32 2292 2298 10.1161/hs1001.097108 11588316

[B18] Suarez JI Qureshi AI Yahia AB Parekh PD Tamargo RJ Williams MA Ulatowski JA Hanley DF Razumovsky AY Symptomatic vasospasm diagnosis after subarachnoid hemorrhage: evaluation of transcranial Doppler ultrasound and cerebral angiography as related to compromised vascular distribution Crit Care Med 2002 30 1348 1355 10.1097/00003246-200206000-00035 12072693

[B19] Lindegaard KF Nornes H Bakke SJ Sorteberg W Nakstad P Cerebral vasospasm after subarachnoid haemorrhage investigated by means of transcranial Doppler ultrasound Acta Neurochir Suppl (Wien) 1988 42 81 84 3055838 10.1007/978-3-7091-8975-7_16

[B20] Vergouwen MD Vermeulen M van Gijn J Rinkel GJ Wijdicks EF Muizelaar JP Mendelow AD Juvela S Yonas H Terbrugge KG Macdonald RL Diringer MN Broderick JP Dreier JP Roos YB Definition of delayed cerebral ischemia after aneurysmal subarachnoid hemorrhage as an outcome event in clinical trials and observational studies: proposal of a multidisciplinary research group Stroke 2011 41 2391 2395 20798370 10.1161/STROKEAHA.110.589275

[B21] Molinaro AM Simon R Pfeiffer RM Prediction error estimation: a comparison of resampling methods Bioinformatics 2005 21 3301 3307 10.1093/bioinformatics/bti499 15905277

[B22] R Development Core Team R: A Language and Environment for Statistical Computing R Foundation for Statistical Computing 2011 Vienna, Austria http://www.r-project.org

[B23] Kassell NF Sasaki T Colohan AR Nazar G Cerebral vasospasm following aneurysmal subarachnoid hemorrhage Stroke 1985 16 562 572 10.1161/01.STR.16.4.562 3895589

[B24] Martin NA Doberstein C Zane C Caron MJ Thomas K Becker DP Posttraumatic cerebral arterial spasm: transcranial Doppler ultrasound, cerebral blood flow, and angiographic findings J Neurosurg 1992 77 575 583 10.3171/jns.1992.77.4.0575 1527618

[B25] Grolimund P Weber M Seiler RW Reulen HJ Time course of cerebral vasospasm after severe head injury Lancet 1988 1 1173 2896998 10.1016/s0140-6736(88)91995-2

[B26] Compton JS Teddy PJ Cerebral arterial vasospasm following severe head injury: a transcranial Doppler study Br J Neurosurg 1987 1 435 439 10.3109/02688698708999633 3077273

[B27] von Holst H Ericson K Haberbeck-Modesto M Steiner L Angiographic investigation of cerebral vasospasm in subarachnoid haemorrhage due to arteriovenous malformation Acta Neurochir (Wien) 1988 94 129 132 10.1007/BF01435865 3213630

[B28] Weir B Grace M Hansen J Rothberg C Time course of vasospasm in man J Neurosurg 1978 48 173 178 10.3171/jns.1978.48.2.0173 624965

[B29] Gonzalez NR Boscardin WJ Glenn T Vinuela F Martin NA Vasospasm probability index: a combination of transcranial doppler velocities, cerebral blood flow, and clinical risk factors to predict cerebral vasospasm after aneurysmal subarachnoid hemorrhage J Neurosurg 2007 107 1101 1112 10.3171/JNS-07/12/1101 18077946

[B30] Sen J Belli A Albon H Morgan L Petzold A Kitchen N Triple-H therapy in the management of aneurysmal subarachnoid haemorrhage Lancet Neurol 2003 2 614 621 10.1016/S1474-4422(03)00531-3 14505583

[B31] Suhardja A Mechanisms of disease: roles of nitric oxide and endothelin-1 in delayed cerebral vasospasm produced by aneurysmal subarachnoid hemorrhage Nat Clin Pract Cardiovasc Med 2004 1 110 116 quiz 2, page following 116 10.1038/ncpcardio0046 16265315

[B32] Hashimoto T Young WL Prohovnik I Gupta DK Ostapkovich ND Ornstein E Halim AX Quick CM Increased cerebral blood flow after brain arteriovenous malformation resection is substantially independent of changes in cardiac output J Neurosurg Anesthesiol 2002 14 204 208 10.1097/00008506-200207000-00005 12172292

[B33] Brown RD Jr Wiebers DO Torner JC O'Fallon WM Frequency of intracranial hemorrhage as a presenting symptom and subtype analysis: a population-based study of intracranial vascular malformations in Olmsted Country, Minnesota J Neurosurg 1996 85 29 32 10.3171/jns.1996.85.1.0029 8683279

[B34] Brown RD Jr Wiebers DO Forbes G O'Fallon WM Piepgras DG Marsh WR Maciunas RJ The natural history of unruptured intracranial arteriovenous malformations J Neurosurg 1988 68 352 357 10.3171/jns.1988.68.3.0352 3343606

[B35] Choi JH Mast H Sciacca RR Hartmann A Khaw AV Mohr JP Sacco RL Stapf C Clinical outcome after first and recurrent hemorrhage in patients with untreated brain arteriovenous malformation Stroke 2006 37 1243 1247 10.1161/01.STR.0000217970.18319.7d 16614321

[B36] Hartmann A Mast H Mohr JP Koennecke HC Osipov A Pile-Spellman J Duong DH Young WL Morbidity of intracranial hemorrhage in patients with cerebral arteriovenous malformation Stroke 1998 29 931 934 10.1161/01.STR.29.5.931 9596237

[B37] van Beijnum J Lovelock CE Cordonnier C Rothwell PM Klijn CJ Al-Shahi Salman R Outcome after spontaneous and arteriovenous malformation-related intracerebral haemorrhage: population-based studies Brain 2009 132 537 543 19042932 10.1093/brain/awn318

[B38] Mohr JP Moskowitz AJ Stapf C Hartmann A Lord K Marshall SM Mast H Moquete E Moy CS Parides M Pile-Spellman J Al-Shahi Salman R Weinberg A Young WL Estevez A Kureshi I Brisman JL The ARUBA trial: current status, future hopes Stroke 2010 41 e537 540 10.1161/STROKEAHA.110.580274 20634478 PMC2927344

[B39] Sloan MA Alexandrov AV Tegeler CH Spencer MP Caplan LR Feldmann E Wechsler LR Newell DW Gomez CR Babikian VL Lefkowitz D Goldman RS Armon C Hsu CY Goodin DS Assessment: transcranial Doppler ultrasonography: report of the Therapeutics and Technology Assessment Subcommittee of the American Academy of Neurology Neurology 2004 62 1468 1481 15136667 10.1212/wnl.62.9.1468

[B40] Bederson JB Connolly ES Jr Batjer HH Dacey RG Dion JE Diringer MN Duldner JE Jr Harbaugh RE Patel AB Rosenwasser RH Guidelines for the management of aneurysmal subarachnoid hemorrhage: a statement for healthcare professionals from a special writing group of the Stroke Council, American Heart Association Stroke 2009 40 994 1025 10.1161/STROKEAHA.108.191395 19164800

[B41] Carrera E Schmidt JM Oddo M Fernandez L Claassen J Seder D Lee K Badjatia N Connolly ES Jr Mayer SA Transcranial Doppler for predicting delayed cerebral ischemia after subarachnoid hemorrhage Neurosurgery 2009 65 316 323 discussion 323-324 10.1227/01.NEU.0000349209.69973.88 19625911

[B42] McMahon CJ McDermott P Horsfall D Selvarajah JR King AT Vail A The reproducibility of transcranial Doppler middle cerebral artery velocity measurements: implications for clinical practice Br J Neurosurg 2007 21 21 27 10.1080/02688690701210539 17453770

[B43] Friedlander RM Clinical practice. Arteriovenous malformations of the brain N Engl J Med 2007 356 2704 2712 10.1056/NEJMcp067192 17596605

